# Yan Hui and Zhuangzi: the way of spiritual healing in the twin peaks of Confucianism and Daoism

**DOI:** 10.3389/fpsyg.2026.1770947

**Published:** 2026-06-22

**Authors:** Junhong Zhu

**Affiliations:** Department of Philosophy and Sociology, Hebei University, Baoding, China

**Keywords:** cognitive transformation, moral cultivation, spiritual well-being, Yan Hui, Zhuangzi

## Abstract

Yan Hui and Zhuangzi have often been read as representing two contrasting traditions of self-cultivation in early Chinese philosophy. Against the background of contemporary concern with psychological distress, this article argues that the *Analects* and the *Zhuangzi* illuminate a shared problem: how the heart-mind may be reoriented under conditions of suffering, instability, and attachment. Through close textual analysis, the article shows that the Confucian materials associated with Yan Hui address this problem by giving desire, conduct, and affect an ethical form through ritual discipline, moral commitment, and reflective self-cultivation, whereas the Daoist materials in the *Zhuangzi* address it by loosening evaluative fixation through stillness, receptivity, and release from rigid conceptual insistence. What emerges is neither a simple juxtaposition of two traditions nor their reduction to a single synthesis, but a more precise theoretical relation: two distinct yet complementary pathways of psychological self-transformation organized around the same pressure on the heart-mind. On this basis, the article proposes an integrative dual-path model in which Confucian moral anchoring and Daoist cognitive release become intelligible as different responses to a shared human difficulty. By reconstructing these pathways from within early Chinese thought itself, the study offers a more explicit account of heart-mind transformation that speaks both to classical Chinese philosophy and to broader theoretical discussions of psychological adjustment and self-cultivation.

## 1 Introduction

According to the World Mental Health Report 2022, more than one billion people worldwide, approximately one-eighth of the global population, are affected by mental disorders. Among them, over 350 million individuals suffer from depression and around 264 million experience anxiety. The World Health Organization further predicts that by 2030 depression may become the leading contributor to the global burden of disease (World Health Organization [WHO], 2022). These developments underscore the urgency of contemporary mental health challenges and the need for broader conceptual resources for thinking about psychological distress.

In recent decades, ideas drawn from Asian philosophical traditions have attracted increasing attention in psychological and clinical discussion. This development suggests that traditions of self-cultivation may offer not only practical inspiration but also alternative theoretical vocabularies for understanding psychological transformation. At the same time, it raises a methodological difficulty. Classical traditions can easily be reduced to premature equivalents of modern therapeutic models, so that their conceptual distinctiveness disappears precisely at the moment they are invoked.

Early Chinese philosophy offers important resources for addressing this difficulty because it develops sustained reflection on the cultivation of the heart-mind (*xin*) and the regulation of emotion, conduct, and awareness. In this context, *xin* does not merely denote a cognitive faculty equivalent to the modern notion of “mind,” but refers more broadly to an integrated center of moral responsiveness, affective life, and practical orientation. At the same time, the article does not claim that the *Analects* develops an explicit theory of *xin* in the more fully articulated sense found in later texts such as the *Mencius*. Here, “heart-mind” is used in a reconstructive and analytic sense to refer to the integrated moral, affective, and practical center implied by the *Analects*’ discussions of joy, anger, learning, ritual discipline, aspiration, and the regulation of the self. The term therefore functions not as a claim about explicit terminological systematization in the *Analects*, but as a disciplined heuristic for comparing two traditions of self-transformation without reducing either to modern psychological vocabulary. Within this field of reflection, the teachings associated with Yan Hui in the Confucian lineage and the philosophical vision articulated in the *Zhuangzi* present two markedly different approaches to heart-mind transformation. The Confucian practice of *ke ji fu li* emphasizes ritual discipline, moral commitment, and the stabilization of the self through ethical orientation. By contrast, the Daoist practices of *xinzhai* and *zuowang* explore ways of loosening rigid conceptual and evaluative attachments so that a more open and responsive mode of awareness may emerge.

Work on the *Analects* has illuminated ethical cultivation, embodied practice, and the ordering of the heart-mind (Slingerland, 2003; Van Norden, 2002), while scholarship on the *Zhuangzi* has highlighted cognitive transformation, perspectival shift, and freedom from evaluative fixation (Ziporyn, 2020). Lin’s account of Confucian learning is especially helpful for the present study because it shows that learning in the *Analects* concerns aspiration, self-cultivation, and the formation of the person rather than the acquisition of information alone (Lin, 2017, pp. 1180–1191). Ko’s reading of the *Zhuangzi* is similarly suggestive because it brings stories of skill and stories of self-cultivation into a single discourse of embodiment, thereby showing that bodily practice, awareness, *qi*, and spirit are not easily separable in Daoist cultivation (Ko, 2025, pp. 2–6). Lai and D’Ambrosio further make clear that Confucianism and Daoism need not be approached as wholly sealed systems, but as partially overlapping fields of reflection on self-cultivation and ways of living (D’Ambrosio, 2020, pp. 2–4; Lai, 2015, pp.489, 492–493, 505, 510). The issue pursued in this article grows out of that terrain: how the relation between these traditions becomes theoretically intelligible when approached through the shared problem of heart-mind transformation rather than through doctrinal alignment alone.

The present study addresses that problem by locating the relation between Confucianism and Daoism in a shared problem-space: how the heart-mind may be reoriented under conditions of suffering, instability, and attachment. Rather than using Western psychological theories simply to validate classical Chinese thought, the article reconstructs two indigenous pathways of psychological self-transformation from within early Chinese philosophy itself. Its comparison of Yan Hui and Zhuangzi does not rest on historical symmetry. Yan Hui is selected as a concentrated Confucian case in which disciplined learning, ritual practice, and moral steadfastness converge; Zhuangzi is selected because his text articulates a Daoist pathway centered on stillness, perspectival release, and freedom from rigid attachment. Their comparability lies not in sameness of status, genre, or doctrine, but in the fact that each makes visible a distinct response to the same problem of heart-mind transformation.

This choice also requires a methodological clarification. The present study does not treat Yan Hui as the author of an independent textual corpus comparable in scale or discursive range to the *Zhuangzi*. Rather, the Confucian side of the comparison is reconstructed from *Analects* materials centered on Yan Hui, where moral steadfastness, disciplined learning, ritual restraint, and affective composure appear in especially concentrated form. The comparison is therefore not between two equivalent authors, but between two exemplary loci through which different pathways of heart-mind transformation become visible. For the purposes of the present argument, Yan Hui is selected not because he is textually symmetrical with Zhuangzi, but because he provides the most focused Confucian case for tracing a pathway of ethical stabilization under pressure.

On this basis, the article proposes an integrative dual-path framework in which Confucian moral cultivation and Daoist cognitive release function as distinct yet complementary mechanisms of response. The Confucian pathway, exemplified by Yan Hui, seeks stability through moral anchoring, ritual discipline, and ethical commitment. The Daoist pathway, exemplified by Zhuangzi, seeks release through contemplative stillness, self-decentering, and the loosening of evaluative fixation. Their relation is therefore best understood not as doctrinal fusion, but as structured complementarity: two different transformational logics addressing the same human difficulty from different philosophical orientations.

The analysis proceeds across four interconnected dimensions: inner cultivation, adversity, embodied regulation, and cognitive-affective transformation. Each dimension contributes a distinct layer to the same theoretical model. Taken together, they are intended to show not simply that Confucianism and Daoism differ, but how their differences become theoretically intelligible within a shared framework of heart-mind transformation.

## 2 Inner cultivation and the reorientation of the heart-mind

The first dimension of the present study concerns inner cultivation as the initial reorientation of the heart-mind. The question here is how confusion, impulse, and attachment are addressed before they harden into more enduring forms of suffering and fixation. At this level, Confucianism and Daoism are not simply two traditions placed side by side, but two distinct ways of responding to the same practical difficulty. The Confucian pathway seeks to reorder the heart-mind by giving desire, conduct, and affect a stable ethical form through ritual discipline and moral commitment. The Daoist pathway seeks to loosen the heart-mind from rigid conceptual and evaluative attachment through stillness, emptiness, and openness to transformation. The contrast reaches beyond ritual and quietude to a deeper divergence in transformational logic: one stabilizes through ethical ordering, while the other releases through cognitive de-fixation. This dimension establishes the basic structure of the article’s dual-path model.

### 2.1 Inner cultivation: ethical discipline and cognitive release

Inner cultivation is the point at which the article’s central contrast first becomes visible in a concentrated way. The issue here is how the heart-mind is worked upon before suffering hardens into confusion, impulsiveness, or attachment. In *the Analects*, especially in passages associated with Yan Hui, transformation begins with the disciplined reshaping of desire, conduct, and affect through ritual practice and self-restraint (Slingerland, 2003). In the *Zhuangzi*, the point of departure is different: the pressure of conceptual insistence and evaluative fixation must be lightened so that awareness can recover openness and responsiveness (Ziporyn, 2020). The contrast therefore concerns more than two styles of self-cultivation. It concerns two different ways of making the heart-mind inhabitable. One proceeds by giving life an ethical form; the other by clearing the obstructions that make awareness contract around itself. The difference established here will continue to shape the later discussions of adversity, embodied life, cognition, and affect.

#### 2.1.1 Confucian inner cultivation through “self-restraint and return to ritual” (ke ji fu li)

The Yan Yuan chapter of the *Analects* preserves one of the most concentrated statements of Confucian self-cultivation. When Yan Hui asks about *ren*, Confucius answers, in Slingerland’s translation, “Restraining yourself and returning to the rites (keji fuli 克己复礼) constitutes Goodness”(Slingerland, 2003, p.125). The weight of this exchange lies in the fact that cultivation begins with the reordering of one’s own dispositions. Desire, attention, speech, and conduct are not left in their immediate form. They are taken up, disciplined, and brought under a normative pattern. What is sought is a heart-mind capable of steadiness because it has acquired moral shape. The present discussion therefore does not treat Yan Hui as the bearer of an independent textual doctrine comparable to the *Zhuangzi*. Rather, it reconstructs a Confucian pathway from *Analects* materials centered on Yan Hui, where ritual discipline, affective restraint, and moral steadfastness appear in especially concentrated form.

Zhu Xi’s gloss remains helpful here. He explains that *ke* means overcoming, *ji* refers to one’s selfish desires, *fu* means returning, and *li* names the patterned articulation of heavenly principle (Zhu, 2011, p. 125). Read this way, *ke ji fu li* does not describe mere outward conformity to ceremonial rule. It names a labor of self-transformation. Personal impulse is checked, not for the sake of repression alone, but so that life may return to a more intelligible order. Ritual provides that order. It gives desire a measure, conduct a form, and affect a direction. The significance of *li* therefore lies as much in inner regulation as in external decorum.

Confucius makes this practical point explicit in the injunction that follows: “Do not look unless it is in accordance with ritual; do not listen unless it is in accordance with ritual; do not speak unless it is in accordance with ritual; do not move unless it is in accordance with ritual”(Slingerland, 2003, p.125). The body’s avenues of engagement with the world are all implicated. Sight, hearing, speech, and action are trained together. Nothing in this passage suggests withdrawal from concrete life. The discipline is enacted amid ordinary perception and conduct. That is why *ke ji fu li* is so important for the present argument. It shows a path of transformation grounded in repetition, restraint, and embodied ethical practice rather than in contemplative emptying. Moral order is built by giving form to response.

Yan Hui’s portrayal in the Analects gives this discipline a human shape. Confucius praises him as one who, in Slingerland’s (2003) translation, “never misdirected his anger and never made the same mistake twice” (p. 53). Zhu (2011) underscores the practical force of the line by explaining that anger, though it may arise, is not redirected onto another object, and that once a fault is recognized it is not repeated (p. 82). The portrait is morally admirable, but it is not merely praise. It discloses a repeatable discipline of affect. Emotion is neither denied nor indulged. It is held, examined, and prevented from expanding into further disorder.

Confucian learning reinforces the same pattern. The *Analects* describes the cultivated person as one who “set your heart upon the Way, rely upon Virtue, lean upon Goodness, and explore widely in your cultivation of the arts”(Slingerland, 2003, p.65). The Mencius adds the familiar reminder that “If in one’s actions one does not succeed, one should always seek for it in oneself” (Van Norden, 2008, p. 91). These texts do not reduce cultivation to isolated acts of self-control. They present an ongoing discipline in which aspiration, reflection, ritual participation, and self-correction work together. Conduct becomes more measured because the heart-mind has learned to return upon itself and accept formation.

Ott and Lai both show that early Confucian cultivation cannot be reduced to inward conviction alone. Embodied practice, patterned action, and the shaping of dispositional life are integral to the formation of the person (Lai, 2015, pp. 505–507; Ott, 2017, pp. 69–73, 80–82). That observation helps clarify what is at stake in Yan Hui’s example. He matters here because he makes visible a concentrated Confucian logic of transformation. The self is steadied by submitting impulse to ritual form, and affect is made durable by moral discipline. The result is not emotional numbness or abstract moralism, but a cultivated coherence of conduct, feeling, and value.

What begins with *ke ji fu li* is therefore not a narrow rule-bound practice, but a structured mode of psychological self-transformation.

#### 2.1.2 Daoist inner cultivation through *Xinzhai* and *Zuowang*

The *Zhuangzi* approaches inner cultivation by changing the condition of awareness itself. Its concern is not how desire can be given ritual form, but how the heart-mind can be freed from the habits that make it cramped, reactive, and prematurely fixed. Ordinary perception and judgment easily harden into possessive involvement with the world. Names, distinctions, intentions, and expectations all begin to press too heavily upon experience. The practices gathered under *xinzhai* and *zuowang* address that burden by easing the grip of conceptual and evaluative insistence.

One of the most influential statements appears in the *Ren Jian Shi* chapter. There Confucius, speaking inside Zhuangzi’s literary construction, introduces *xinzhai*. Ziporyn translates the passage as follows:

“You have so single-mindedly focused your will that you have been constantly hearkening to it, not with your ears but with your mind, and not only with your mind but even with your vital energy. Instead let your hearkening stay positioned at the ears, your mind going no further than meshing there like a tally. The vital energy is then a vacuity, a waiting for the presence of whatever thing may come. The Course alone is the gathering of this vacuity. This vacuities the fasting of the mind” (Ziporyn, 2020, p.37).

The progression matters. Ear, mind, and *qi* are not simply ranked as better and worse faculties. What emerges is a criticism of ordinary consciousness when it rushes to “match things up,” classify, and take hold. Vacuity here does not mean vacancy in the sense of lifeless absence. It means a cleared condition in which awareness no longer forces things into rigid patterns before they can appear on their own terms.

That is why *xinzhai* should not be reduced to quietism. It does not merely recommend calmness, and it does not celebrate passivity for its own sake. The point lies in receptivity. Once the heart-mind ceases to impose itself so aggressively, another mode of responsiveness becomes possible. Experience is no longer governed at every moment by fixed conceptual sorting or evaluative urgency. The world is encountered with less interference. What changes is not external reality, but the manner in which reality is allowed to arrive within awareness.

The *Da Zong Shi* chapter carries this movement further in the account of *zuowang*. Yan Hui says, “It’s a dropping away of my limbs and torso, a chasing off of my sensory acuity, dispersing my physical form and ousting my understanding until I am the same as the Transforming Openness. This is what I call just sitting and forgetting”(Ziporyn, 2020, p.62). The rhetoric is radical, but the target is precise. What is being loosened is attachment to the bodily, sensory, and intellectual markers through which the self habitually secures its identity. The passage does not recommend stupidity or self-annihilation. It describes a release from overdetermined self-consciousness. Awareness no longer clings so tightly to what it takes itself to be.

Guo Xiang’s commentary helps illuminate the force of this move. He explains that *zuowang* involves forgetting not only traces, but that by which traces are traced, so that inwardly one is no longer confined within the body and outwardly no longer arrested by the separation of Heaven and Earth; one then becomes vastly open and identified with transformation itself (Guo, 2011, p. 152). The importance of this comment lies in its refusal to treat forgetting as simple subtraction. What falls away is a rigid structure of fixation. What emerges is openness. The heart-mind is not emptied into blankness, but released into a less possessive way of dwelling amid change.

The Zhuangzi may be read as reconfiguring the way selfhood is inhabited and related to the world. What is at issue is not simply a contrast between sincerity and pretense, but a more fundamental loosening and reformation of the self through emptiness (Machek, 2016, pp.52–54, 58–59). That line of interpretation is useful here because it clarifies what *xinzhai* and *zuowang* accomplish. The issue is not the negation of awareness, but a loosening of the compulsions through which awareness becomes cramped, possessive, and overdetermined. Perception becomes less grasping, judgment less rigid, and response less defensive. What these practices make possible is a different mode of inhabiting the self rather than the disappearance of selfhood altogether. Cultivation proceeds by release rather than by normative shaping.

The contrast with the Confucian path is sharp, but it should not be flattened into a simple opposition between discipline and spontaneity. Both traditions are confronting the same difficulty: the heart-mind does not remain clear, steady, or free on its own. It becomes burdened by habit, impulse, and attachment. The Daoist answer takes that burden up by loosening fixation at its source. *Xinzhai* clears the conditions of receptivity; *zuowang* carries the clearing into the very structure of self-awareness. Together they disclose a second path of inner cultivation, one in which transformation proceeds through cognitive release.

Read together, these two modes of practice show how the heart-mind may be transformed either through ethical formation or through the release of fixation. The next section turns to hardship and adversity, where the consequences of these orientations become easier to see.

### 2.2 Adversity and transformation: moral endurance and cognitive release

Hardship brings the difference between these two orientations into sharper relief. Once deprivation, frustration, and instability are no longer treated in the abstract, the question is no longer simply how one cultivates oneself, but how one lives through pressure without being inwardly broken by it. The Confucian materials centered on Yan Hui show adversity being taken up within an ethical horizon, so that difficulty becomes a test of steadfastness and a site of self-formation. The *Zhuangzi* turns attention elsewhere. It asks how suffering gains its force from the standards by which loss, failure, and injury are judged. One path preserves significance under pressure; the other reduces the tyranny of pressure by loosening the hold of evaluative fixation. Seen from this angle, adversity does not merely confirm a prior distinction between Confucianism and Daoism. It reveals what those different practices of the heart-mind amount to when life becomes genuinely difficult.

#### 2.2.1 Preserving ethical commitment in times of material deprivation

Material deprivation occupies a prominent place in the Analects’ portrayal of Yan Hui, but the significance of these descriptions does not lie in treating poverty as something admirable in itself. What the *Analects* preserves is a more difficult claim, external hardship need not overthrow the heart-mind when ethical commitment has become sufficiently stable. The point is not that deprivation ceases to be deprivation, nor that suffering is somehow unreal. What changes is the relation between hardship and inner orientation. Poverty no longer dictates the worth of one’s life, because worth is anchored elsewhere.

The best-known statement appears in the *Yong Ye* chapter. Confucius praises Yan Hui in these words: “What a worthy man was Yan Hui! Living in a narrow alley, subsisting on a basket of grain and gourd full of water—other people could not have born such hardship, yet it never spoiled Hui’s joy. What a worthy man was Hui!”(Slingerland, 2003, p. 56). The sentence becomes trivial if read as praise of temperament alone. Its real force lies in the contrast between objective deprivation and the persistence of an undiminished inner orientation. Others “could not have born such hardship” the situation because they remained governed by the ordinary relation between material condition and affective state. Yan Hui does not cease to inhabit the same conditions, but their power over the heart-mind has changed.

Zhu Xi’s commentary gives that contrast added precision. He notes that Confucius praises Yan Hui not because hardship has somehow disappeared, but because despite the extremity of his situation, Hui remains settled and undisturbed (Zhu, 2011, p. 85). What is being admired is not passivity, nor a dullness to suffering, but the preservation of moral composure where ordinary persons would become inwardly disordered. Deprivation presses upon the body and upon circumstance, yet it does not dislodge the ethical center of the person.

That center is supplied by *ren* and the life organized around it. A person whose deepest orientation depends upon wealth, recognition, or comfort will experience their loss as a collapse of significance. A person whose life is already ordered by ethical aspiration stands in a different relation to misfortune. For Yan Hui, the pursuit of *ren* provides a criterion of worth that is not reducible to material success. That is why hardship can become a site of testing without becoming a site of inner ruin. The heart-mind remains under pressure, but it does not become captive to pressure.

Li argues that the joy associated with Confucius and Yan Hui should not be reduced to mood or subjective pleasure, because it is inseparable from a morally formed way of life (Li, 2021, pp. 145–152). That observation is crucial here. The joy in question is not the pleasant feeling of someone who has escaped difficulty. It is the durable steadiness of someone whose evaluative center has already been educated. Yan Hui’s example therefore shows how adversity may be borne without surrender when moral commitment has become affectively constitutive.

A limited comparison with Frankl may be helpful. Logotherapy proposes that suffering is lived differently when it is held within a horizon of meaning (Frankl, 2018, pp. 116–119). The analogy should not be pressed too far. In the Confucian case, meaning is not generated by subjective projection alone, but arises from participation in an ethical tradition shaped by *ren*, ritual practice, and self-cultivation. The comparison remains useful because it helps illuminate what is happening in Yan Hui’s case, where suffering does not vanish but its place within life is reorganized.

The larger importance of this passage lies in the form of transformation it reveals. Material deprivation remains fully real, yet it no longer has final authority over the heart-mind. Ethical commitment becomes the medium through which hardship is held, interpreted, and endured. The result is not resignation, nor emotional anesthesia, but a strengthened coherence of value and affect. What is visible here is a distinctly Confucian way of passing through adversity, in which difficulty becomes the occasion on which the depth of moral anchoring is disclosed.

#### 2.2.2 Value transformation under conditions of individual setback

Setback in the *Zhuangzi* is rarely treated as a purely external event. What gives adversity its oppressive force is the network of judgments through which a situation is taken as humiliating, deficient, useless, or failed. Loss is experienced not only because something happens, but because the heart-mind remains tightly bound to the standards by which that happening is measured. The *Zhuangzi* therefore does not begin by asking how one may remain morally steadfast amid misfortune. It asks what sort of evaluative posture turns misfortune into psychic constriction in the first place.

The famous theme of “how useful uselessness is” (Ziporyn, 2020, p. 43) makes this point with particular clarity. In the *Ren Jian Shi* chapter, the sacred oak appears worthless from the perspective of conventional utility. Precisely for that reason, however, it escapes being cut down and is able to preserve its life and grow to fullness (Ziporyn, 2020, p. 41). The force of the image lies in the instability it introduces into accepted standards of value. A thing judged useless from one standpoint may prove profoundly beneficial from another. What had seemed to mark deficiency now becomes the condition of preservation.

The same logic is also visible in the figure of “Take Outspread the Discombobulated” (Ziporyn, 2020, p.42). Ordinary social judgment takes his body as a sign of deficiency and social insignificance. Yet the very features that render him “useless” in the eyes of the world protect him from conscription, labor, and exploitation, allowing him to remain outside the machinery that consumes ordinary lives (Ziporyn, 2020, p. 42). The point is not that deformity is secretly desirable, nor that all social distinctions can simply be reversed. The *Zhuangzi* is exposing the narrowness of the standards by which ordinary agents sort worth from worthlessness. What counts as failure is often inseparable from the evaluative system that first defined the field of competition.

Nelson argues that the *Zhuangzi* repeatedly draws attention to the malformed, the marginal, and the apparently useless in order to unsettle instrumental judgments of value (Nelson, 2014, pp. 732–733). That observation is important here because it helps show that the issue is not paradox for paradox’s sake. The text is investigating the psychic consequences of evaluative rigidity. Once utility becomes the sole criterion of worth, whatever falls outside it is experienced as defect. Once that criterion is loosened, what had been lived as inadequacy can be inhabited differently.

This is why setback in the Daoist context takes on a distinctive shape. What changes is not merely one’s emotional reaction to a fixed situation, but the situation’s status within the horizon of value. The heart-mind ceases to grant unquestioned authority to prevailing distinctions of achievement and failure. Distress loses some of its force because the standards that made the situation unbearable no longer dominate experience in the same way. One does not have to deny pain in order to loosen its hold. A change in evaluative orientation is enough to alter how pain is borne.

A cautious analogy with Adler may illuminate this movement. Adler’s reflections on inferiority and the creative self suggest that what appears as limitation may be reworked through a different practical orientation (Adler, 2017, pp. 26–27). The comparison is only partial. The *Zhuangzi* is not recommending compensatory self-assertion, nor a heroic conversion of weakness into strength. Its movement is at once more radical and quieter, loosening the evaluative field in which the opposition between superiority and inferiority has become psychologically binding.

Read alongside the previous section on Yan Hui, the difference between these two responses to adversity becomes more precise. Confucian cultivation preserves significance by holding fast to an ethical center under pressure. Daoist cultivation reduces the tyranny of pressure by weakening the standards through which loss and setback seize the heart-mind. One path deepens endurance through moral commitment; the other lightens suffering through value transformation. Their divergence becomes most visible when hardship is no longer considered abstractly, but as something lived from within.

Taken together, these two sections bring the second major dimension of the article into view. Adversity does not produce a single kind of transformation. In one case, hardship discloses the depth of moral anchoring; in the other, hardship becomes livable because its evaluative framework has shifted. The difference matters because it shows that the dual-path model is not a verbal pairing of two traditions, but an account of two genuinely distinct ways in which the heart-mind may be altered under pressure. The next part of the article turns from adversity to embodied life, where these orientations reappear in the shaping of bodily conduct, vitality, and the relation between body and heart-mind.

## 3 Nourishing life and embodied regulation: two pathways of harmonizing body and heart-mind

The third dimension of the present study concerns embodied regulation. The issue here is not simply how life is preserved, but how bodily practice participates in the ordering or release of the heart-mind. This dimension is necessary to the dual-path model because neither Confucian nor Daoist self-transformation is purely “mental” in a modern disembodied sense. In the Confucian case, bodily conduct is disciplined so that ethical attention, restraint, and reverence may become stable and repeatable. In the Daoist case, bodily quietude and natural attunement reduce interference so that openness, flexibility, and responsiveness may re-emerge. What makes these pathways comparable, then, is not that they share a single doctrine of nourishing life, but that both treat the body as a practical medium of heart-mind transformation. This dimension specifies how the dual-path model extends from ethical and cognitive processes into embodied life.

### 3.1 Nourishing life as embodied self-cultivation: ethical discipline and natural attunement

The question of nourishing life shifts the discussion from hardship to the more sustained problem of how life is kept inhabitable over time. Early Chinese thought does not treat this question as a matter of bodily survival alone. What is at stake is a way of living in which body, vitality, conduct, and inward orientation can remain in workable relation. The Confucian materials approach that relation through discipline, measure, and the gradual acquisition of durable form. The Daoist materials approach it through easing obstruction, restoring rhythm, and learning how not to force life away from its own movement. The contrast matters because it extends the argument beyond explicit self-cultivation or adversity response. It shows that the divergence between these traditions reaches into the practical maintenance of life itself.

#### 3.1.1 Confucian nourishing life through ethical self-discipline

Discussions of nourishing life in the Confucian tradition rarely begin from specialized techniques for prolonging bodily existence. What stands in the foreground is the ordering of life through moral cultivation. The body is neither ignored nor celebrated as an autonomous object of care. It is gathered into a larger discipline in which conduct, affect, intention, and ethical aspiration gradually come into alignment. Life is preserved, sustained, and made stable through the formation of the person.

Yan Hui provides an instructive example because his cultivation is described not in terms of physical technique, but in terms of enduring moral concentration. Confucius remarks that “Ah, Yan Hui! For 3 months at a time his heart did not stray from Goodness”(Slingerland, 2003, p. 55). The importance of this remark lies in the continuity it implies. The heart-mind does not flash briefly toward the good and then fall back into disorder. It acquires a certain settledness. That settledness bears directly upon life as lived. A person whose inner orientation is repeatedly scattered by impulse, resentment, or excess cannot easily sustain bodily and emotional balance. A person whose life is held together by moral discipline inhabits the body differently.

This is where ritual becomes indispensable. In the *Analects*, *li* disciplines seeing, hearing, speaking, and bodily movement by bringing them under measure and restraint (Slingerland, 2003, p. 125). This regulation is not merely external. It changes the tempo of response, so that impulse no longer governs the person immediately. The body learns restraint, and restraint, repeated over time, alters disposition. What is preserved through such practice is more than propriety in the narrow sense. A stable rhythm of life begins to emerge.

The broader Confucian tradition makes the same point in more systematic form. The *Book of Rites* records detailed prescriptions for posture, gesture, gaze, breath, voice, and comportment (Sun, 2022, p. 791). These are easy to dismiss as formalism if read apart from the larger logic of cultivation. Yet their very minuteness reveals what Confucian thought is after. Bodily life is not left to chance. The way one stands, sits, looks, speaks, and breathes all contribute to the shaping of the person. Order enters not only through explicit moral decision, but through repeated bodily practice. A life governed by measure becomes less vulnerable to inner dissipation.

Scholarship on embodied knowledge in Confucianism helps clarify what is at stake. Ott argues that early Confucian cultivation should be understood through practices that form the person bodily as well as mentally, rather than through belief or doctrine alone (Ott, 2017, pp. 65–66, 69–73, 80–82). That insight is especially useful here. Nourishing life in the Confucian sense does not require a sharp division between bodily care and moral discipline. The two belong together. Ethical self-discipline carries bodily consequences because it regulates appetite, moderates excess, and stabilizes the rhythms through which life is actually lived.

This point should not be overstated into a claim that Confucianism possesses a developed physiological theory of health comparable to later medical or Daoist traditions. The emphasis lies elsewhere. The preservation of life is approached through the cultivation of an ordered person. Moral aspiration is not added to life after the fact. It becomes the shape through which life avoids becoming chaotic, dissipated, or inwardly divided. The value of Yan Hui’s example lies precisely here. His life shows how ethical discipline may become a sustaining force at the level of heart-mind, conduct, and daily existence.

What this section makes visible is a specifically Confucian way of linking nourishment and order. Life is steadied when the person acquires durable form. Bodily existence is not mastered by technical intervention, but supported by the repeated integration of value, affect, and conduct. In that sense, nourishing life here belongs to the same movement already traced in the earlier parts of the article. Life becomes inhabitable because moral anchoring has become strong enough to hold it together.

#### 3.1.2 Daoist nourishing life through natural attunement and stillness

The *Zhuangzi* speaks of nourishing life from a noticeably different sensibility. What threatens life is not only hardship from outside, but the accumulation of forcing, striving, friction, and misalignment within one’s way of inhabiting the world. A life becomes damaged when it is pressed too hard into purposes alien to its own movement. Preservation therefore depends less on regulating the person through normative form than on easing obstruction and learning how to remain in accord with what is already unfolding.

The chapter *Yang Sheng Zhu* gives the classic formulation. Ziporyn (2020) translates the line as follows: “For it tends toward the current of the empty central meridian as its normal route. And this is what enables us to maintain our bodies, to keep the life in them intact, to nourish those near and dear to us, and to fully live out our years” (p.29). The wording does not describe a dramatic intervention into life. It points instead toward continuity, guidance, and adherence to an inner course. Turner’s interpretation is useful here because it shows that the *Zhuangzi*’s concern with *yangsheng* cannot be reduced to techniques of preservation alone, but is bound up with the practical activities through which life is sustained and lived (Turner, 2023, pp. 50–55, 60–62). Read in this way, nourishing life is not simply a matter of extending bodily duration. It concerns the maintenance of a workable relation between body, activity, and the conditions under which life unfolds. What must be avoided is not only physical harm, but the kinds of forcing and misalignment that make life inwardly unlivable.

The story of Cook Ding belongs to the same horizon. When he first cut up oxen, he saw the whole animal before him. After years of practice, he no longer relied upon ordinary visual effort alone, but came to encounter the ox with what Ziporyn calls “the imponderable spirit in me rather than scrutinizing it with the eyes”(Ziporyn, 2020, p.30). Ko’s reading of the *Zhuangzi* helps sharpen this point by treating stories of skill and stories of self-cultivation together as a coherent discourse of embodiment in which body, heart, *qi*, and spirit work in coordinated relation rather than as separate domains of practice (Ko, 2025, pp. 1–4). Cook Ding matters in that context not simply because he is skillful, but because his bodily responsiveness no longer tears against the grain of things. Skill becomes philosophically significant when it discloses what life looks like after force has yielded to attunement.

The *Ke Yi* chapter develops this orientation more explicitly through breathing and guiding exercises. In Ziporyn’s translation, the line reads: “Breathing in and out with puffs and roars, spitting out the old breath to take in the new, hanging like a bear and stretching like a bird, concerning themselves only with health and long life” (Ziporyn, 2020, p. 128). The phrase is notable for its practical concreteness. Breath, movement, and bodily extension all belong to the work of sustaining life. Traditional commentaries explain these actions in terms of guiding *qi*, regulating breath, and softening bodily tension (Guo, 2013, p. 478). The important point for the present discussion is less the technical detail than the larger pattern. Preservation is sought through rhythm, circulation, and release from blockage.

Stillness gives this pattern its deepest form. The phrase “Consider the gaps and cracks and hollows in things: it is in the empty chambers that light appears, and all auspicious things come to roost only where there is stillness” in the *Ren Jian Shi* chapter suggests that clarity appears when inner obstruction recedes (Ziporyn, 2020, pp. 37–38). Quiet is not pursued here as an end in itself. It makes room. It lessens the crowding of perception, intention, and worry. Cheng Xuanying’s explanation emphasizes that emptiness in the heart-mind allows true illumination to arise of itself (Guo, 2011, p. 79). That remark helps show why stillness is inseparable from nourishment. A life harried by excess inward activity becomes depleted. A life capable of returning to openness regains room to breathe, move, and respond.

Daoist stillness is best understood here as an embodied mode of cultivation rather than as abstract quietude. The Zhuangzi does not treat body and heart-mind as separate domains requiring separate therapies. Tension in one reverberates in the other. When striving, fixation, and disturbance are eased, bodily life becomes more inhabitable. Nourishing life thus names a practical art of reducing unnecessary friction between vitality, awareness, and the world one moves within.

The difference from the Confucian orientation now comes into clearer focus. There the sustaining force of life appeared through ethical self-discipline and the acquisition of durable form. Here life is sustained by recovering a more yielding relation to process, movement, and change. One path supports life by strengthening moral coherence; the other supports life by reducing the violence of overcontrol. Both are concerned with how life may remain viable, but they answer that concern through very different habits of body and heart-mind.

Read together, these two sections complete another necessary layer of the article’s argument. The question of nourishment shows that the divergence between the two traditions extends beyond doctrine or emotional style into the practical maintenance of life itself. One way of living becomes steadier by accepting form, measure, and discipline; another becomes more livable by easing strain, restoring rhythm, and making room for transformation. The contrast matters because it prepares the transition to the next part of the article, where body and heart-mind will no longer be considered only in terms of life-preservation, but in terms of the concrete mechanisms through which bodily practice reshapes awareness, affect, and response.

### 3.2 Embodied mechanisms of body–heart-mind transformation

Once nourishing life has been considered in broader philosophical terms, a further question presses forward: through what concrete bodily processes does transformation actually take place? The body cannot remain a vague background to inward cultivation. It is one of the places where the difference between the two traditions becomes materially visible. Confucian texts emphasize patterned conduct, ritualized posture, and disciplined bodily action, all of which shape the heart-mind by giving response a stable form. Daoist texts place greater weight on quieting, softening, and reducing interference, so that bodily life becomes less strained and awareness less rigid. What emerges in this section is not a secondary supplement to the earlier argument, but a more precise account of how practice takes hold in lived experience. The contrast between ethical ordering and release from overconstraint must finally be traced in the body itself.

#### 3.2.1 Confucian embodied regulation through Li (ritual propriety)

Within the Confucian tradition, the cultivation of the heart-mind is inseparable from the disciplined shaping of bodily conduct through *li* (ritual propriety). This connection is central to the embodied dimension of the present study because it shows that bodily regulation is not secondary to moral cultivation, but one of its primary means. The Confucian pathway does not treat the body as a merely external vehicle for an already formed inward morality. Rather, the body becomes a practical site in which ethical attention, restraint, and reverence are enacted and stabilized. In this respect, the embodied dimension of Yan Hui’s cultivation remains theoretically comparable to the Daoist alternative, even though the two traditions differ sharply in how bodily practice functions.

The normative shaping of bodily conduct receives systematic expression in Confucian ritual texts. The *Book of Rites*, in the *Yu Zao* chapter, prescribes that “the feet should be placed in a measured way, the hands held in reverence, the eyes kept straight, the mouth kept closed, the voice quiet, the head upright, the breath solemn, the stance proper, the expression composed, and the sitting posture still” (Sun, 2022, p. 791). These prescriptions are not merely ceremonial details. They show that Confucianism understands posture, gesture, speech, and sensory restraint as practical disciplines through which inner order is gradually cultivated. The body is shaped so that the heart-mind may become attentive, restrained, and ethically responsive.

The same logic is visible in the Analects account of *ke ji fu li*, where ritual extends beyond public conduct to the regulation of perception, speech, and bodily movement (Slingerland, 2003, p. 125). What repeated practice produces here is a form of embodied moral attention. At the level of mechanism, bodily discipline carries ethical normativity into stable habits of seeing, hearing, speaking, and acting.

Ott’s account of embodied knowledge makes clear that early Confucian cultivation cannot be reduced to inward belief alone, but is inseparable from embodied practice and the disciplined formation of the person (Ott, 2017, pp. 65–66, 69–73, 80–82). Read in this light, Confucian embodied regulation works through moral anchoring at the level of bodily comportment. The point is not simply that ritual expresses inner virtue once virtue is already there, but that ritualized conduct helps produce the bodily and affective conditions under which moral stability becomes possible.

#### 3.2.2 Daoist embodied release through stillness and natural attunement

Daoist embodied practice begins from a different concern. What requires transformation is not bodily conduct through normative form, but the strain and interference that keep body and heart-mind from settling into a more open relation. The point is not bodily neglect, but a different understanding of how body and heart-mind interact. In the Zhuangzi, bodily stillness and natural attunement do not merely preserve life in a physiological sense; they reduce interference, loosen fixation, and restore a more open responsiveness to transformation. In this respect, Daoist embodied practice becomes the bodily counterpart to cognitive release.

A concentrated expression of this orientation appears in the Zhuangzi’s descriptions of cultivated stillness. In the *Qiwulun* chapters, the ideal state is rendered through the striking phrase “Can the body really be made like a withered tree, the mind like dead ashes” (Ziporyn, 2020, p. 11). The image does not recommend numbness, lethargy, or bodily collapse. What it depicts is the suspension of excessive intention, agitation, and cognitive overactivity. The body is no longer driven into restless engagement, and the heart-mind is no longer crowded by reactive involvement. Stillness matters here because it interrupts the mutual reinforcement of bodily tension and mental fixation.

This is why Daoist embodied cultivation should be understood in terms of release rather than discipline. When bodily movement is quieted and sensory urgency reduced, entrenched patterns of perception and reaction begin to loosen. The practitioner is not trained into an externally imposed order; rather, excessive bodily and cognitive activity gradually falls away. In that sense, stillness functions as a practical mode of de-intensification. It reduces the friction through which body and heart-mind keep one another in states of strain, distraction, and overdetermination.

Daoist stillness may be understood as an embodied and experiential mode of cultivation rather than as abstract mystical speculation. That formulation is especially useful here because it clarifies that bodily quietude is not secondary to inner transformation, but one of the places where transformation occurs. The Zhuangzi does not treat body and heart-mind as separate domains requiring separate therapies. Tension in one reverberates in the other. When striving, fixation, and disturbance are eased, bodily life becomes more inhabitable and awareness less rigid. Embodied release names that process more precisely than stillness taken in isolation.

The broader philosophical force of this pathway lies in the fact that bodily quietude becomes a gateway to cognitive flexibility. Once external agitation subsides, the heart-mind is no longer compelled to cling so tightly to fixed judgments and oppositions. Awareness regains room to respond without being driven by habitual insistence. This is the embodied side of what the present study has called cognitive release. The body is not shaped into ethical order, as in the Confucian case, but brought into a quieter, less resistant relation with process, rhythm, and change.

Read together with the previous section, this contrast clarifies the third major dimension of the article. Confucian embodied regulation works by forming bodily conduct through ritual measure, so that ethical attention and restraint become stable in practice. Daoist embodied release works by easing strain and reducing interference, so that fixation weakens and responsiveness becomes less constrained. The difference should not be mistaken for a contrast between bodily cultivation and bodily neglect. What it reveals instead is that early Chinese thought developed two distinct embodied pathways through which the relation between body and heart-mind could be transformed.

## 4 Cognitive transformation and emotional cultivation: two pathways of heart-mind transformation

The fourth dimension of the present study concerns cognition and affect: how the heart-mind is no longer governed by confusion, fixation, or emotional disturbance. At this level, the dual-path model becomes especially explicit. The Confucian pathway seeks stability through disciplined learning, moral reflection, and ethical commitment; the Daoist pathway seeks release through perspectival loosening, conceptual de-fixation, and a wider horizon of awareness. These are not merely two attitudes toward thought and feeling, but two different ways of reorganizing the conditions under which cognition and emotion arise. In the Confucian case, cognition, emotion, and conduct are progressively ordered into a morally intelligible whole. In the Daoist case, cognitive and affective disturbance are weakened by loosening the standpoint from which rigid distinctions and evaluative entanglements are sustained. This dimension therefore completes the article’s dual-path framework by showing how each pathway reconfigures cognition and affect at the level of heart-mind process.

### 4.1 Complementary cognitive pathways: disciplined understanding and cognitive release

The cognitive dimension brings the article back to the heart-mind in a more focused way. What sort of understanding steadies a life, and what sort of understanding frees it from fixation? The Confucian materials associated with Yan Hui place weight on learning, reflection, and the gradual extension of insight through disciplined study. The *Zhuangzi* is concerned with another difficulty: cognition can become trapped within the very forms it uses to grasp the world. Knowledge then stiffens into compulsion. The opposition here is not one between thinking and non-thinking, nor between knowledge and anti-intellectualism. It concerns two different relations to understanding itself. One deepens and orders it; the other loosens the hold it acquires when it hardens into rigidity.

#### 4.1.1 Confucian cognitive formation through disciplined learning

Yan Hui’s learning matters here because it shows how understanding may become a mode of heart-mind formation rather than the mere acquisition of information. The significance of his example lies not simply in the praise of unusual giftedness, but in the fact that his learning displays a distinctive Confucian mechanism of cognitive stabilization in which knowledge, reflection, and moral cultivation progressively reinforce one another. In this sense, the Confucian cognitive pathway differs from the Daoist one not because it values cognition while Daoism abandons it, but because it seeks transformation through disciplined understanding rather than through the loosening of conceptual mediation.

Confucius repeatedly presents Yan Hui in precisely these terms. In the *Wei Zheng* chapter of the *Analects*, he remarks, in Slingerland’s (2003) translation, “I can talk all day long with Yan Hui without him once disagreeing with me. In this way, he seems a bit stupid. And yet when we retire and I observe his private behavior, I see that it is in fact worthy to serve as an illustration of what I have taught. Hui is not stupid at all” (p.11). What is emphasized here is not passive receptivity, but the capacity to internalize teaching, reflect upon it, and extend it through one’s own understanding. Yan Hui’s apparent quietness therefore marks not intellectual deficiency, but disciplined assimilation.

A similar point appears in the well-known characterization of Yan Hui as one who “learns one thing and thereby understands ten” (Slingerland, 2003, p. 42). The significance of this remark lies not simply in quickness of mind, but in the fact that learning here becomes cumulative and expansive. One insight becomes the basis for a wider pattern of judgment. At the level of mechanism, this suggests a mode of cognition in which repeated learning gradually generates interpretive range, moral clarity, and psychological steadiness. Read through the framework of the present study, the Confucian pathway works here through cognitive stabilization rather than cognitive release.

This cognitive pattern is closely connected with the learning trajectory reflected in *the Analects* passage where Confucius says, “I am not bitter toward Heaven, nor do I blame others. I study what is below in order to comprehend what is above” (Slingerland, 2003, p. 168). Zhu Xi glosses this as the movement from concrete human affairs toward the comprehension of moral principle, explaining that “to study human affairs below is precisely the way to reach heavenly principle above” (Zhu, 2011, p. 148). The point is that Confucian cognition does not oppose everyday practice to higher understanding. Rather, concrete experience becomes the disciplined basis from which moral insight gradually takes shape. What is formed through this process is not abstract knowledge alone, but a stable orientation in which cognition, value, and self-cultivation are progressively integrated.

Lin argues that learning in Confucius is not reducible to book knowledge, but is oriented toward aspiration, self-cultivation, and the formation of one’s proper self (Lin, 2017, pp. 1180–1181, 1189–1190). This helps clarify why Yan Hui’s learning matters so much here. What appears in his case is not the accumulation of information, but the gradual shaping of an inward orientation in which reflection, value, and practical life increasingly cohere. Understanding itself becomes a mode of self-formation. Knowledge is not merely accumulated; it is ordered into a morally intelligible structure of the heart-mind. Disciplined learning thus stabilizes the heart-mind by progressively integrating knowledge, reflection, and ethical orientation.

#### 4.1.2 Daoist cognitive release through intuitive insight

The Zhuangzi turns the question of cognition in another direction. The problem is not ignorance alone, but the way knowledge hardens into forms that constrain awareness. What is at issue here is not the rejection of cognition as such, but a different understanding of how cognition becomes free. In the *Zhuangzi*, insight does not arise primarily through cumulative ordering, but through release from the forms of knowing that have become constraining. This is why the Daoist side of the present study is best described not as anti-intellectual negation, but as cognitive release.

A central expression of this view appears in the *Wai Wu* chapter of the *Zhuangzi*. In Ziporyn’s translation, the passage reads: “A fish trap is there for the fish. When you get the fish, you forget the trap. A snare is there for the rabbits. When you get the rabbit, you forget the snare. Words are there for the intent. When you get the intent, you forget the words”(Ziporyn, 2020, p. 224). The point is not that language and concepts are useless, but that they are instrumental rather than ultimate. Once understanding has been achieved, attachment to the means of understanding becomes an obstacle rather than a help. Read in relation to the Confucian pathway, this indicates a sharply different cognitive logic: what must be transformed is not ignorance alone, but fixation on the very forms through which understanding is ordinarily pursued.

The same logic is radicalized in the practice of zuowang. As Guo Xiang’s commentary makes especially clear, what is at issue is not the elimination of awareness, but the release of the heart-mind from its rigid dependence on bodily attachment, sensory control, and discursive intellect (Guo, 2011, p. 152). In this sense, zuowang names a process in which the heart-mind becomes free not by accumulating more conceptual structure, but by loosening its dependence on structure once that structure has hardened into constraint.

Wong reads Zhuangzi’s treatment of self and awareness not as a simple elimination of cognition, but as a transformation in the mode of relation between self and world (Wong, 2021, pp. 245–246, 251–252, 259–260). What emerges here is therefore a distinct psychological mechanism. When habitual conceptual control is relaxed, the heart-mind becomes capable of a more flexible and less possessive form of understanding. The result is not ignorance, but freedom from rigid knowing. If Yan Hui’s pathway stabilizes the heart-mind through disciplined understanding, Zhuangzi’s pathway releases it by loosening attachment to conceptual form. What emerges instead is a form of understanding freed from rigid conceptual insistence.

Comparisons with modern cognitive psychology may offer a limited interpretive aid at this point. Research on insight, for example, suggests that understanding may sometimes emerge through reorganization rather than through linear step-by-step reasoning (Jung-Beeman et al., 2004, p. e97). Such comparison should remain secondary, however. Its value lies only in clarifying, by analogy, how release from habitual organization may open the possibility of sudden comprehension; it does not provide the theoretical basis of the Daoist account.

### 4.2 Affective transformation: moral reorientation and emotional release

Emotional disturbance brings the stakes of heart-mind transformation into especially immediate view. Pain, frustration, anger, and sorrow are never merely inner events detached from value; they arise within ways of interpreting the world and one’s place in it. The Confucian texts respond by drawing feeling back into an ethical horizon, where suffering can be borne, clarified, and reoriented through moral commitment. The *Zhuangzi* follows another route. It attends to the ways feeling is intensified by possessive judgment, reactive involvement, and rigid distinctions, then seeks relief by loosening that grip. The contrast here is exacting. One path gives emotion a more durable moral shape; the other lightens the pressure that makes emotion oppressive. What follows will show how these different treatments of affect complete the larger picture of heart-mind transformation developed across the article.

#### 4.2.1 Confucian affective reorientation through moral narrative

Emotional disturbance in the Confucian tradition is not treated as something to be eliminated outright. What requires transformation is not feeling as such, but the way feeling is situated within the life of the heart-mind. Early Confucian thought does not separate cognition, emotion, and volition into independent faculties. Rather, *xin* names the living center in which moral awareness, affective responsiveness, and practical orientation converge. For that reason, emotional cultivation does not proceed through detachment from value, but through the ethical clarification of feeling itself. Distress becomes bearable, and even transformative, when it is gathered into a morally intelligible horizon. Here, “heart-mind” is used in an analytic rather than a strictly terminological sense. The point is not to claim that the *Analects* articulates an explicit theory of *xin* comparable to later texts such as the *Mencius*, but to name the integrated moral-affective-practical center implied by its discussions of joy, anger, learning, ritual discipline, and self-regulation.

A clear example appears in the *Wei Ling Gong* chapter of the *Analects*. When Confucius and his disciples face hardship during their travels, Zilu shows visible frustration. Confucius responds, in Slingerland’s translation, that “the gentleman encounters hardship” (Slingerland, 2003, p. 174). The force of this response lies not in telling Zilu to suppress distress, nor in denying the severity of the situation. What changes is the meaning of the situation. Confucius relocates hardship within an ethical horizon in which steadfastness, rather than immediate relief, becomes the relevant measure of response. Emotion is not negated; it is reoriented.

This same logic is concentrated in the descriptions of joy associated with Confucius and Yan Hui. In the *Yong Ye* chapter, Confucius praises Yan Hui in these words: “What a worthy man was Yan Hui! Living in a narrow alley, subsisting on a basket of grain and gourd full of water—other people could not have born such hardship, yet it never spoiled Hui’s joy” (Slingerland, 2003, p. 56). In the *Shu Er* chapter, Confucius likewise remarks, “Eating plain food and drinking water, having only your bent arm as a pillow—certainly there is joy to be found in this!” (Slingerland, 2003, p. 69). These passages do not celebrate deprivation for its own sake. Nor do they describe joy as an emotional residue left over after suffering has been denied. What they show is that material hardship need not determine the state of the heart-mind when moral commitment provides a more enduring center of orientation. Zhu Xi makes this point succinctly when he says that such joy is not derived from something externally pleasurable, but arises from within the cultivated self (Zhu, 2011, p. 85).

The emotional mechanism at work here may be described as moral reorientation through narrative and value. Confucian thought does not leave feeling at the level of immediate reaction. Emotion is interpreted, guided, and gradually reshaped through *ren*, *li*, and the moral vocabulary of self-cultivation. One does not step outside evaluative life; one learns to inhabit it more rightly. Scholarship on early Chinese philosophy has likewise shown that emotion in these traditions is not merely a private mental state but part of the larger relation between self, world, and cultivated response (Virág, 2017, pp. 4–5,196–197). The point, then, is not simple emotional restraint, but the formation of an affective life capable of bearing suffering without surrendering moral intelligibility.

Modern emotion theory offers a limited analogy here. Lazarus’s account of appraisal suggests that emotional response depends not only on events themselves but on how those events are interpreted (Lazarus, 1991, pp. 123–145). The comparison should not be pressed too far. Confucian affective transformation is grounded not in subjective reframing alone, but in the ethical work of the heart-mind as cultivated through aspiration, ritual discipline, and moral commitment. Emotional disturbance is thus transformed not by loosening value, but by bringing feeling into a more stable and morally ordered relation to value.

#### 4.2.2 Daoist affective release through the loosening of evaluative attachment

Zhuangzi approaches emotional disturbance from a very different angle. The problem is not that human beings feel too much, but that feeling becomes bound to rigid judgments, possessive identifications, and narrow distinctions that the heart-mind takes too seriously. Suffering is intensified when events are immediately absorbed into oppositions such as gain and loss, success and failure, self and other. What Daoist cultivation seeks is not moral reinterpretation of those experiences, but a loosening of the standpoint that makes them psychically binding in the first place. Emotional freedom begins when the grip of evaluative entanglement weakens.

The “empty boat” (Ziporyn, 2020, p.159) passage in the *Shan Mu* chapter provides one of the clearest illustrations of this point. As rendered by Ziporyn, anger does not arise merely from collision as such, but from the attribution of agency and intention: when the boat is empty, even an irritable person does not become enraged; when it is taken to be occupied, anger immediately follows (Ziporyn, 2020, p. 159). The passage does not recommend numbness or emotional indifference. Its point is subtler. Once the event is no longer taken as a personal affront issuing from an intentional other, the emotional charge changes. What has shifted is not the external situation alone, but the stance from which the situation is apprehended. Anger loses force because reactive involvement loses force.

A broader version of the same affective logic appears in the *Xiao Yao You* chapter. Through the image of the *kunpeng* rising ninety thousand *li* into the sky, Zhuangzi opens a perspective beyond the cramped standards by which ordinary agents measure worth, achievement, and loss. Ziporyn translates the passage as “chariot upon what is true both to Heaven and to earth, riding atop the back-and-forth of the six atmospheric breaths” (Ziporyn, 2020, p. 5). This is not an escape from the world into abstraction. It is a release from the narrow evaluative frameworks that make ordinary life oppressive. *Xiaoyao* names a condition in which the heart-mind is no longer overdetermined by rigid distinctions or external standards. Guo Xiang’s commentary moves in the same direction when it explains that things differ in size and capacity, yet each may still attain its own fulfillment when allowed to follow its own nature (Guo, 2011, p. 2). Freedom here is not the absence of feeling, but the easing of the habits that make feeling constricted and burdensome.

Wong reads Zhuangzi’s account of selfhood and freedom not as aiming at emotional deadening, but at a transformation in the mode of awareness through which the self relates to the world (Wong, 2021, pp. 245–247, 257–260). That point helps clarify the distinct mechanism at work here. Emotional suffering is attenuated not because hardship disappears, but because the standpoint through which events are taken as personally binding no longer governs the heart-mind with the same force. One becomes less defensive, less possessive, and less rigidly invested in distinction. This is what the present article means by affective release through the loosening of evaluative attachment.

Contemporary psychotherapy offers a limited analogy. Dialectical Behavior Therapy encourages an observing stance toward emotion rather than total identification with it (Linehan, 1993, pp. 230–231, 369–370). The comparison is useful only up to a point. Daoist affective cultivation is not grounded in therapeutic technique, but in a transformed relation between self, judgment, and world. Suffering may therefore be reduced not only by being given ethical meaning, but also by loosening the evaluative entanglements that make it so difficult to bear.

## 5 Discussion

**FIGURE 1:**
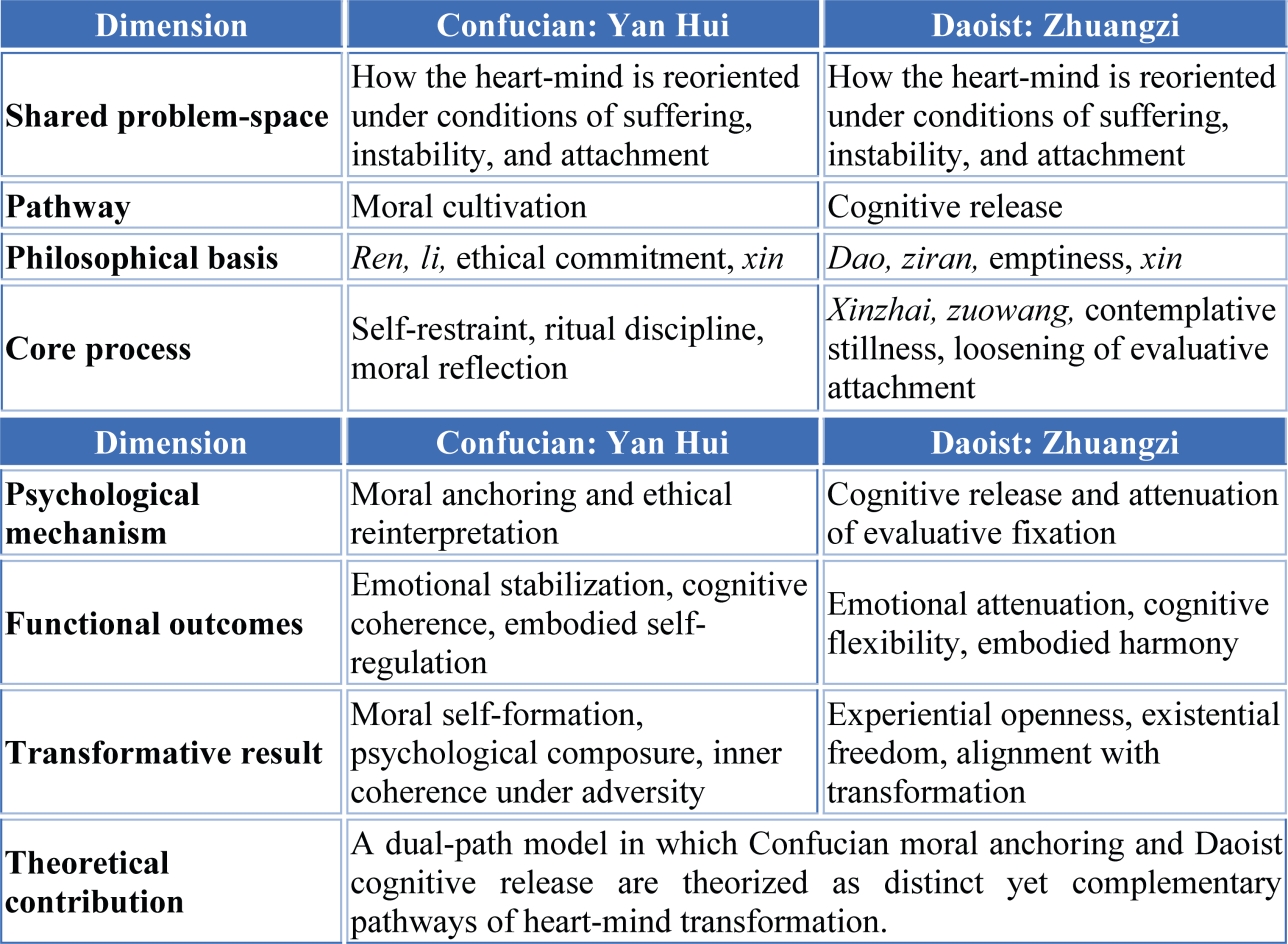
An integrative dual-path model of heart-mind transformation in Confucianism and Daoism.

The therapeutic orientations represented by Yan Hui and Zhuangzi within the Confucian and Daoist traditions offer culturally grounded resources for rethinking psychological distress without reducing classical Chinese thought to a precursor of modern therapy. Rather than using contemporary psychological theories simply to validate early Chinese philosophy, the present study has reconstructed two indigenous pathways of psychological self-transformation from within the traditions themselves. Lai and D’Ambrosio show that Confucianism and Daoism need not be treated as wholly sealed systems, but can be approached as partially overlapping fields of reflection on self-cultivation and ways of living (Lai, 2015,489–490, 504–506; D’Ambrosio, 2020, 1–4). Ko’s account of embodiment and Machek’s account of transformed selfhood are especially relevant here because they make it difficult to treat Daoist self-cultivation as merely anti-Confucian or anti-ethical in spirit (Ko, 2025, pp. 1–4; Machek, 2016, pp. 52–54, 58–60). Their work helps clarify the comparative space in which the present argument moves. The relation between the two traditions becomes clearer when their differences are read as differently organized responses to the same pressure on the heart-mind, rather than as a problem of whether they can be merged into a single framework. Building on that insight, the present article has argued that their relation becomes theoretically intelligible when located within a shared problem-space: how the heart-mind is reoriented under conditions of suffering, instability, and attachment. Figure 1 presents this framework in schematic form.

Figure 1 makes explicit the article’s central claim. The point is not simply that Confucian and Daoist cultivation differ, but that they become theoretically comparable at the level of a shared problem-structure while remaining distinct at the level of transformational mechanism. This is what allows the study to move beyond interpretive juxtaposition. Rather than placing two traditions side by side, it shows how each responds to the same difficulty of heart-mind reorientation through a different mechanism of response.

The contrast is clearest when traced across the four dimensions examined in the article. On the Confucian side, ritual discipline, moral commitment, reflective learning, and ethical reinterpretation converge in the stabilization of the self. On the Daoist side, stillness, perspectival loosening, embodied attunement, and affective release converge in the weakening of fixation and the recovery of openness. Yan Hui and Zhuangzi become analytically comparable here because each makes visible a different pathway of heart-mind transformation within the same problem-space.

A further contribution of the model is that it clarifies the internal coordination of body, cognition, and affect within each pathway. On the Confucian side, disciplined learning, moral reflection, ritualized bodily conduct, and ethical reinterpretation converge in the formation of a stable and morally ordered heart-mind. On the Daoist side, contemplative quietude, perspectival loosening, embodied attunement, and affective release converge in the weakening of fixation and the recovery of responsiveness. In this respect, the article contributes not merely a comparison of two traditions, but a more explicit account of how distinct modes of self-cultivation can be theorized as internally coherent responses to the same problem of psychological self-transformation.

At the same time, the argument has clear limits. It does not claim that Yan Hui and Zhuangzi exhaust the full range of Confucian and Daoist approaches to self-cultivation, nor that later developments in these traditions can be reduced to the two pathways identified here. Nor does it claim direct empirical verification for the efficacy of these pathways in a modern clinical sense. Nor does it claim that the Confucian side rests on an independent Yan Hui corpus; rather, it reconstructs a Confucian pathway from *Analects* materials centered on Yan Hui as a concentrated exemplary case.

Further work may develop this model in two directions. One is theoretical: clarifying more precisely how indigenous notions of the heart-mind may contribute to contemporary discussions of self-regulation, cognition, and affect without collapsing their philosophical specificity (Virág, 2017, 1–5, 17–24). The other is interdisciplinary: exploring whether selected elements of Confucian ethical reflection or Daoist contemplative practice bear measurable relations to resilience, meaning-making, or cognitive flexibility. In either case, the present argument is best understood not as a final synthesis, but as a conceptually disciplined framework for further dialogue.

Early Confucianism and Daoism thus emerge here as two distinct yet complementary pathways of heart-mind transformation within a shared human problem-space.

## Data Availability

The original contributions presented in the study are included in the article/supplementary material, further inquiries can be directed to the corresponding author.
